# Antibiotic dosing in the 'at risk' critically ill patient: Linking pathophysiology with pharmacokinetics/pharmacodynamics in sepsis and trauma patients

**DOI:** 10.1186/1471-2253-11-3

**Published:** 2011-02-20

**Authors:** Jason A Roberts, Michael S Roberts, Andrew Semark, Andrew A Udy, Carl MJ Kirkpatrick, David L Paterson, Matthew J Roberts, Peter Kruger, Jeffrey Lipman

**Affiliations:** 1 Burns, Trauma and Critical Care Research Centre, The University of Queensland, Royal Brisbane and Women's Hospital, Brisbane, Queensland, Australia; 2 Department of Intensive Care Medicine, Royal Brisbane and Women's Hospital, Brisbane, Queensland, Australia; 3 Pharmacy Department, Royal Brisbane and Women's Hospital, Brisbane, Queensland, Australia; 4 School of Medicine, The University of Queensland, Brisbane, Queensland, Australia; 5 School of Pharmacy and Medical Sciences, University of South Australia, Adelaide, Australia; 6 School of Pharmacy, The University of Queensland, Brisbane, Queensland, Australia; 7 Departments of Infectious Diseases and Microbiology, Royal Brisbane and Women's Hospital, Herston, Brisbane, Australia; 8 University of Queensland Centre for Clinical Research, Brisbane, Australia; 9 Department of Intensive Care Medicine, Princess Alexandra Hospital

## Abstract

**Background:**

Critical illness, mediated by trauma or sepsis, can lead to physiological changes that alter the pharmacokinetics of antibiotics and may result in sub-therapeutic concentrations at the sites of infection. The first aim of this project is to identify the clinical characteristics of critically ill patients with significant trauma that have been recently admitted to ICU that may predict the dosing requirements for the antibiotic, cefazolin. The second aim of this is to identify the clinical characteristics of critically ill patients with sepsis that may predict the dosing requirements for the combination antibiotic, piperacillin-tazobactam.

**Methods/Design:**

This is an observational pharmacokinetic study of patients with trauma (cefazolin) or with sepsis (piperacillin-tazobactam). Participants will have samples from blood and urine, collected at different intervals. Patients will also have a microdialysis catheter inserted into subcutaneous tissue to measure interstitial fluid penetration of the antibiotic. Participants will be administered sinistrin, indocyanine green and sodium bromide as well as have cardiac output monitoring performed and tetrapolar bioimpedance to determine physiological changes resulting from pathology. Analysis of samples will be performed using validated liquid chromatography tandem mass-spectrometry. Pharmacokinetic analysis will be performed using non-linear mixed effects modeling to determine individual and population pharmacokinetic parameters of antibiotics.

**Discussion:**

The study will describe cefazolin and piperacillin-tazobactam concentrations in plasma and the interstitial fluid of tissues in trauma and sepsis patients respectively. The results of this study will guide clinicians to effectively dose these antibiotics in order to maximize the concentration of antibiotics in the interstitial fluid of tissues.

## Background

The incidence of infection in critically ill patients, suffered before or following admission to ICU, is increasing. In Australia and New Zealand, 12% of admissions to intensive care units (ICU's) are for severe sepsis [1]. Despite advances in critical care medicine, 27% of these patients will die [1]. Improved antibiotic therapy has been proposed as a mechanism to improve outcome for septic patients [2,3], although the achievement of this depends on both the timely selection of an appropriate antibiotic and on using an appropriate dose so as to obtain appropriate antibiotic concentrations at the site of infection.

Data describing the likely benefits of appropriate dosing of antibiotics remains sparse [4-7]. The antibiotic dosing schedule for a trauma patient admitted to ICU is presently poorly defined and largely empiric. Dosing schedules rely largely on data obtained in non-critically ill patients and there is increasing evidence that describes how such an approach is likely to result in inadequate therapy [2,8].

In trauma, tissue damage leads to activation of the innate immune system with the release of cytokines, interleukins and other mediators of inflammation [9]. This response may be localised or systemic; if systemic it is referred to as the systemic inflammatory response syndrome (SIRS). Sepsis is then defined as SIRS in the presence of a suspected or documented infection [10]. Many pathological processes (such as trauma, pancreatitis, burns and major surgery) can produce a SIRS response, leading to sepsis, if an infection is super-imposed. The hemodynamic changes common to the SIRS response, regardless of the initiating insult, include a low systemic vascular resistance and a high cardiac output described as a hyperdynamic circulation [11].

This hyperdynamic circulatory SIRS response has been demonstrated to have an effect on the creatinine clearance (CL_CR_) [12]. The magnitude of this effect is such that CL_CR _can be increased by 50-100% [13]. This increase in CL_CR _can occur in the presence of a normal serum creatinine concentration [14]. Identification of these patients based on laboratory creatinine concentrations is thus difficult. In fact, it has been demonstrated that an isolated serum creatinine concentration within the "normal" reference range is an insensitive indicator of the glomerular filtration rate in the critically ill [15]. The haemodynamic changes, induced by the SIRS response, may thus lead to altered pharmacokinetics (PK) through an augmented renal clearance in patients without significant renal dysfunction [16]. CL_CR _is an important determinant in the pharmacokinetics of antibiotics in the critically ill [17,18].

A significant concern for clinicians is that the administration of standard antibiotic doses in such patients may result in a sub-therapeutic concentration, leading to sub-optimal bacterial kill. In trauma patients receiving antibiotic prophylaxis, this may increase the risk of sepsis. In a patient with sepsis this may result in treatment failure with an increased mortality [2,3,19]. In addition, in both trauma and septic patients, failure to achieve adequate concentrations may result in the development of antibiotic resistance [20]. As such identification of the physiological characteristics of patients with a hyperdynamic circulation and thus an augmented renal clearance is poorly described and clinically important.

The optimal measurement of renal function in critically ill patients is still uncertain [16]. The Cockcroft-Gault equation is still widely utilized but its application in critically ill patients has been questioned [15,21-23]. Urinary creatinine collections of 8, 12 and 24 hrs have been used (with matched serum creatinine concentrations) although this will tend to overestimate GFR at lower filtration rates. Sinistrin clearance has also been used to measure GFR due to its unique properties of limited renal tubular secretion and reabsorption and may provide insight into the accuracy of other approaches in critically ill patients [24].

In addition to changes in renal function, trauma and sepsis may also induce increased capillary leakage, microvascular stasis and the accumulation of peripheral fluid [11]. Previous studies evaluating antibiotic PK in the presence of sepsis or trauma have shown the volume of distribution of hydrophilic antibiotics to be significantly increased [24-26]. This capillary leak syndrome is well described in sepsis and to a lesser extent in patients with major trauma [27-29]. Trauma is further implicated in altered pharmacokinetics secondary to possible reduced intravascular volume, massive fluids shifts, and hypoproteinemia [3,30]. Hypoproteinemia is well described in sepsis and critical care [31]. These changes impact on antibiotic diffusion distance and delivery resulting in slow and possible incomplete antibiotic tissue penetration into infected tissues [3,16,32,33].

A consequence of the physiological changes, seen in both septic and trauma patients, is that these two subgroups of critically ill patients are at risk of significantly different pharmacokinetic (PK) profiles with respect to that of non-critically ill patients. As such standard treatment regimens are likely to be ineffective and result in sub-therapeutic target tissue concentrations.

Currently there is little data to guide the clinician to predict which patients may have different PKs in the acute phase of trauma or during sepsis. Furthermore most studies have examined the total drug concentrations in serum, rather than the pharmacologically active unbound drug in tissues where most infections occur [34]. This uncertainty of dosing requirements, in the presence of data that highlights the importance of appropriate antibiotic therapy, is of critical importance.

The identification of the physiological predictors of altered PK allows the development of dosing algorithms that can maximise the target site antibiotic concentration and reduce treatment failure.

As detailed above there are many causes for potential altered PK in patients both with sepsis or trauma. There is some overlap between the physiological pathways that cause these alterations in PK. However, we propose to study sepsis and trauma patients in separate groups, with the sepsis group receiving treatment with piperacillin-tazobactam and the trauma group receiving prophylaxis with cefazolin.

Piperacillin-tazobactam is a combination of a semisynthetic penicillin and β-lactamase inhibitor that is commonly used for empiric therapy of sepsis. Cefazolin is a narrower spectrum first generation cephalosporin antibiotic commonly used as prophylaxis in critically ill patients with traumatic injuries. Little data exists to confirm the appropriateness of dosing of these important antibiotics for critically ill patients. There is no data describing potential interrelationships between the kinetics of these antibiotics and the physiological changes that occur in patients that have sepsis or significant traumatic injuries.

## Aims

The first aim of this project is to identify the clinical characteristics of critically ill patients with significant trauma, that have been recently admitted to ICU, that may predict the dosing requirements for the antibiotic, cefazolin.

The second aim of this is to identify the clinical characteristics of critically ill patients with sepsis that may predict the dosing requirements for the combination antibiotic, piperacillin-tazobactam.

Our approach is to accurately describe the patient's altered physiology and develop physiologically-based pharmacokinetic (PBPK) models that may describe plasma and tissue cefazolin and piperacillin-tazobactam concentrations for each of these patient groups.

## Methods/Design

### Study design

This is an open-labelled pharmacokinetic study. Samples of blood, tissue microdialysate and urine will be collected from participants being treated in the ICU of the Royal Brisbane and Women's Hospital (RBWH), Queensland, Australia and the Princess Alexandra Hospital (PAH), Queensland, Australia for sepsis or major trauma.

### Setting

The clinical study will be performed in the ICU at RBWH and the PAH, which have approximately 4000 patients admitted annually. This work will be done in collaboration with the Burns, Trauma and Critical Care Research Centre and the Therapeutics Research Centre, School of Medicine, The University of Queensland.

### Identification of Eligible Patients

Participants would need to meet the inclusion and exclusion criteria to be enrolled. Informed consent will be obtained from each patient or a legally authorised representative to participate in the study.

### Inclusion criteria

• Age of between 18 years and 80 years.

• Clinical indication for therapy with cefazolin for trauma patients or piperacillin-tazobactam after diagnosis of sepsis where sepsis is defined as:

○ Clinical suspicion of infection and/or positive culture results

○ SIRS as defined by two or more of the following [10]:

▪ Core temperature <36°C or >38°C

▪ Tachycardia as defined by a heart rate >90 beats per minute

▪ Tachypnoea as defined by a respiratory rate greater than 20 breaths per minute OR a PaCO_2 _less than 32 mmHg during spontaneous breathing OR the requirement for mechanical ventilation.

▪ A white blood cell count >12 × 10^9^/L or <4 × 10^9^/L OR greater than 10% immature (band) forms.

• Renal function as defined by:

○ Serum creatinine concentration <170 μmol/L

• An arterial line in situ

• Written informed consent obtained

### Exclusion criteria

Participants will be excluded if they have pre-existing renal impairment (serum creatinine concentration ≥171 μmol/L) or if they have a history of allergy to the study antibiotic or iodine.

### Participants

Participants will be recruited into two separate groups:

1. Trauma patients (n = 30) receiving cefazolin; and

2. Sepsis patients (n = 50) receiving piperacillin-tazobactam.

### Drug dosing

Antibiotic administration will only occur in patients where the treating clinician has deemed a clinical need for the study antibiotic. Administration will occur in line with the manufacturer's guidelines for each product. Cefazolin will be administered in 20 ml 0.9% sodium chloride by intravenous infusion via a central venous catheter over 5-minutes and piperacillin-tazobactam will be administered in 50 ml 0.9% sodium chloride by intravenous infusion via a central venous catheter over 20-minutes.

Other marker compounds will also be administered to assess the physiology of the patient at the time of antibiotic administration.

Indocyanine green (ICG) - Pulsion 0.5 mg/kg (maximum dose 50 mg) will be administered to provide a measure of plasma volume, an indication of hepatic function and the distribution kinetics of highly bound solutes. Plasma volume will be obtained from the final ICG concentration [35]. The ICG will be administered as a rapid bolus via central venous line 10 min prior to the start of the antibiotic infusion. ICG non-invasive oximetry measurements will be taken over a period of 7 min.

Sodium bromide (5% w/v solution; 1 ml/kg) will be administered intravenously to measure extracellular fluid fluctuations [36]. It will be administered as a slow bolus over 3 min. For cefazolin, sodium bromide will be administered starting 2 min after the initiation of the antibiotic infusion and for piperacillin-tazobactam, it will be administered for the final 3-minutes of the 20-minute antibiotic infusion [37].

Sinistrin 2500 mg (Inutest^R^, Laevosan, Linz, Austria) will be given as a bolus over 30 seconds to help determine renal function (GFR). For cefazolin, sinistrin will be administered over the final 30-seconds of the antibiotic infusion and for piperacillin-tazobactam, it will be administered over the final 30-seconds of the 20-minute antibiotic infusion [38].

### Sample collection

Sampling will occur during one 6-hour dosing interval for each patient.

#### Blood Samples

Six blood samples will be taken for each antibiotic.

Piperacillin-tazobactam (infused over 20 min) - the first sample will be taken 1 minute prior to start of the infusion and then at 20 min (end of infusion), 40 min, 60 min, 210 min and 360 min respectively post commencement of the infusion.

Cephazolin (infused over 5 min). The first sample will be taken 1 minute prior to the infusion of the antibiotic and then at 5 min (end of infusion), 20 min, 60 min, 210 min and 360 min respectively post commencement of the infusion.

#### Microdialysis Samples

Following insertion of a microdialysis catheter (CMA 60, 20 kDa dialysis window, Global Scientific, Sweden) subcutaneously by an experienced clinician, fourteen samples will be collected over the course of the antibiotic dosing interval. The catheter will be perfused with a solution of 0.9% sodium chloride and cefalotin (10 mg/L) which acts as a marker antibiotic of similar molecular size and physicochemical properties to the study antibiotics. Cefalotin will assist in the determination of the rate of movement of molecules across the dialysis membrane according to the retrodialysis method [39,40]. The first microdialysis sample is taken 1 min prior to the start of the antibiotic infusion with repeat measurements taken at 20 min, 40 min, 60 min, 90 min, 120 min, 150 min, 180 min, 210 min, 240 min, 270 min, 300 min, 330 min, and at 360 min respectively following which the microdialysis catheter will be removed.

#### Urine Samples

Urine samples for creatinine clearance will be collected as previously described according to the urinary collection clearance method [41]. Urine collection will start at the time of initiation of the antibiotic infusion. At 2 hours, 4 hours and 6 hours the urine will be collected with a 5 ml aliquot taken for quantification of the rate and amount of antibiotic excreted in that particular 2 hour time period. After the 6-hour sample, a subsequent 8-hour collection will commence providing four urinary creatinine clearance measures over a 14-hour period.

#### Tetrapolar Bioimpedance

An experienced clinician will perform tetrapolar bioimpedance on two occasions during the study. The first measurement occurring 20 minutes prior to the antibiotic dose and then repeated six and a half hours post initiation of the infusion. The results will be used for measuring the fat free mass [42] of the patient and detecting altered fluid status (e.g. variations in extracellular water) over the study period.

#### Cardiac Output

Pulse contour arterial waveform analysis will be used to measure cardiac output and other derived haemodynamic measurements utilizing the FloTrac/Vigileo™ system (Edwards Lifesciences, Irvine, CA, USA). Cardiac output results will be confirmed using an UltraSonic Cardiac Output Monitor (The USCOM ultrasonic CO monitor USCOM Pty, Coffs Harbour, NSW, Australia). These measurements will be made simultaneously at three times post the initiation of the antibiotic infusion (0 minutes, 180 minutes and 300 minutes).

### Sample handling and storage

Blood samples that are collected will immediately be placed on ice and centrifuged within 60-minutes of sampling at 3000 rpm for 10-minutes and stored at -80°C until assay. Microdialysis and urine samples will be stored at -80°C until assay.

### Sample analysis

Assays of all samples will occur, within 30-days of collection, at the Therapeutics Research Centre of The University of Queensland.

Blood, urine and microdialysis samples will be analysed using validated liquid chromatography-tandem-mass spectrometry (LC-MS/MS) analytical assays. Blood plasma protein and albumin concentrations will be measured. The unbound fraction of antibiotic in plasma will be determined using ultracentrifugation (12000 rpm for 20-minutes) through 3 kDa nominal cut-off membrane devices (Amicon^® ^YM30, Millopore Corporation, Billerica, MA).

ICG, sodium bromide and sinistrin concentrations will all be measured using validated High-Performance-Liquid-Chromatography (HPLC) assays. ICG concentration clearance will also be assessed by non-invasive oximetry (LiMon - Pulsion Medical Systems) Non-invasive tetrapolar bioimpedance will be compared with measured sodium bromide concentrations.

Analysis of urine samples from the eight-hour urinary collection will be compared with other measures of renal function, including results from the sinistrin analysis (glomerular filtration) and creatinine clearance determination using the Cockroft-Gault formula [43].

Analysis of the 5 ml urine aliquots for antibiotic clearance will be performed using validated LC-MS/MS assays and will determine the rate and amount of antibiotic excreted in urine.

### Data collection

Additional data will be obtained from the medical record and will include:

1. Participant demographics including age, gender, height, allergies, co-morbidities;

2. Clinical details (admission diagnosis, progress and outcome);

3. Measures of illness severity, including Acute Physiology and Chronic Health Evaluation (APACHE) II score on admission [44];

4. A daily Sepsis Organ Failure Assessment (SOFA) score [45];

5. Revised Trauma Score (RTS) [46];

6. A tissue penetration and perfusion score consisting of oxygen saturation (%), serum lactate concentration (mmol/L) and noradrenaline dose (mcg/kg/min) will be taken at the time of the antibiotic infusion [47];

7. Microbiology results - Gram stain, culture and antimicrobial sensitivities of blood cultures or other culture sites following admission to hospital;

8. Laboratory investigations - full blood count, serum biochemistry, coagulation profile, liver function tests and arterial blood gas measurements.

### Statistical considerations

It is anticipated that a minimum of 30 patients with data-rich sampling will be required to develop a pharmacokinetic model defining at least 3 predictors. The sample size is based on a power of 80%, a level of significance of 5%, multiple regression analysis with 3 predictor variables and a R-square (proportion of variation explained) of 30% [48].

### Data analysis

Pharmacokinetic modelling of antibiotic distribution and clearances will be performed and compared, where possible, to previously published pharmacokinetic data from healthy volunteers and other non-critically ill patient groups. The results of sample analysis will be analysed using a non-linear mixed effects modelling approach (NONMEM, GloboMax LLC, Hanover, MD, USA). The aim of this process will be to develop a baseline population physiologically-based-pharmacokinetic (PBPK) model for each antibiotic assuming a re-circulatory PBPK-pharmacodynamic (PBPKPD) model [49,50]. NONMEM will mainly be used to model between subject variability and within subject variability in simultaneously modelling sparse data from multiple patients with a classical compartmental approach. Each of the factors that are identified to affect antibiotic pharmacokinetics will then be incorporated into the model to help describe the between subject variability and within subject variability, defining, where possible, the extent a patient's pharmacokinetics changes during critical illness. Incorporation of all variables will be used to develop a user-friendly antibiotic dosing algorithm.

The influence of demographic and clinical covariates will be tested in the model. The model will simulate antibiotic pharmacokinetics for different dosing schedules to predict the best dosing recommendations for sepsis patients receiving piperacillin-tazobactam and trauma patients receiving cefazolin.

### Ethical considerations

The Human Research and Ethics Committee of the RBWH (2007/188). Princess Alexandra Hospital and the Medical Research Ethics Committee of the University of Queensland (2008000449) have approved this study.

### Withdrawal from Study

Participants may withdraw from the study at anytime without prejudice, as documented and explained at the time of consenting.

## Discussion

It is becoming increasingly evident that the dosing requirements for antibiotics in critically ill patients are different to non-critically ill patients. The pharmacokinetics in critically ill patients is affected by changes in haemodynamic parameters, hypoproteinemia, microvascular flow and capillary leak. This is due, in part, to the hyperdynamic circulation and augmented renal clearance induced by the SIRS response in both trauma and sepsis, resulting in increased GFR. The altered tissue physiology found in sepsis and trauma with increased capillary leakage and blood flow stasis may lead to altered volumes of distribution and slow antibiotic penetration. Trauma and sepsis are further implicated in altered pharmacokinetics secondary to possible reduced intravascular volume, massive fluids shifts, and hypoproteinemia. These two specific groups of patients are therefore at risk of altered pharmacokinetics and the potential for antibiotic underdosing demands further investigation.

## Conclusions

Inappropriate antibiotic administration can lead to selection of resistant organisms and failure of therapy with increased mortality [2,3]. Identification of the physiological characteristics that may predict an altered pharmacokinetic profile will enable optimised dosing in these individuals. The proposed study will identify parameters instructive of the need for altered antibiotic doses to ensure therapeutic concentrations in both plasma and in the interstitial fluid of tissues.

## Abbreviations

ICU: intensive care unit; PK: pharmacokinetic; RBWH: Royal Brisbane and Women's Hospital; PAH: Princess Alexandra Hospital; ICG: Indocyanine green; USCOM: UltraSonic Cardiac Output Monitor; LCMSMS: liquid chromatography-mass spectrometry; HPLC: High-Performance-Liquid-Chromatography; PBPKPD: PBPK-pharmacodynamic

## Competing interests

The authors declare that they have no competing interests.

## Authors' contributions

JR, MSR, PK and JL designed the study and wrote the protocol. AS and MR assisted writing the protocol. AU, CMJ, and DLP provided advice and input into the protocol. All authors have read and approved the final manuscript.

## Funding

Funding for this project is provided by the National Health and Medical Research Council of Australia, Project Grant 519702. JR is funded by a Fellowship from the National Health and Medical Research Council of Australia (569917).

## Pre-publication history

The pre-publication history for this paper can be accessed here:

http://www.biomedcentral.com/1471-2253/11/3/prepub
